# A Non-invasive Chromosome Screening Strategy for Prioritizing *in vitro* Fertilization Embryos for Implantation

**DOI:** 10.3389/fcell.2021.708322

**Published:** 2021-08-09

**Authors:** Li Chen, Qin Sun, Juanjuan Xu, Haiyan Fu, Yuxiu Liu, Yaxin Yao, Sijia Lu, Bing Yao

**Affiliations:** ^1^Department of Reproductive Medicine, Affiliated Jinling Hospital, Medicine School of Nanjing University, Nanjing, China; ^2^Department of Medical Statistics, Jinling Hospital, Southern Medical University, Nanjing, China; ^3^Department of Clinical Research, Yikon Genomics Company, Ltd., Suzhou, China

**Keywords:** preimplantation genetic screening, non-invasive chromosome screening, blastocyst, spent culture medium, next generation sequencing

## Abstract

Preimplantation genetic testing for aneuploidy (PGT-A) is widely used to select embryos having normal ploidy for transfer, but they require an invasive embryo biopsy procedure that may cause harm to the embryos and offspring. Therefore, a non-invasive approach to select embryos with normal ploidy for implantation is highly demanded. Non-invasive chromosome screening (NICS) methods have been proposed and applied in clinical practices, but a large-scale validation versus invasive preimplantation genetic testing (PGT) and the whole embryo ploidy has not yet been reported. In this study, by using the whole embryo as a gold standard, we validated NICS assay in a total of 265 donated human embryos and compared its performance with conventional trophectoderm (TE) biopsy PGT. The NICS assay showed promising performance, which is comparable to PGT-TE [sensitivity: 87.36 versus 89.66%; specificity: 80.28 versus 82.39%; negative predictive value (NPV): 91.2 versus 92.86%; positive predictive value (PPV): 73.08 versus 75.73%]. Additionally, NICS provides a scoring system for prioritizing embryo: embryos can be categorized into three groups with euploid prediction probabilities of 90.0, 27.8, and 72.2% for group euploid (A), aneuploid (B), and multiple abnormal chromosomes (MAC) (C), respectively. When an addition of TE assay is provided as a secondary validation, the accuracy significantly increases from 72.2 to 84.3% for group B and from 27.8 to 83.3% for group C. Our results suggest that NICS is a good rule in assay for identifying chromosomal normal embryos for transfer and might serve as a non-invasive approach for prioritizing embryos instead of preventing transfer of aneuploid and MAC embryos. It will help to ensure the safety of offspring and efficient utilization of embryos.

## Introduction

How to prioritize embryo for transfer is the key issue in *in vitro* fertilization (IVF) treatments. Currently, the most commonly used method to assess the developmental potential of an embryo in culture remains to be morphology. Despite its convenience, morphology is a poor indicator of embryo chromosomal composition and is highly subjective. It is possible that embryos with high morphological scores carry genetic defects. With the development of gene sequencing technology, preimplantation genetic testing (PGT) has been widely applied in IVF practices to select embryos. Although multiple clinical trials have demonstrated improved clinical outcomes with preimplantation genetic testing for aneuploidy (PGT-A) (Forman et al., 2013; Scott et al., 2013; Neal et al., 2018), it remains highly controversial whether PGT-A is truly beneficial and should be offered to patients of all ages (Kushnir et al., 2016; Gleicher and Orvieto, 2017). One major concern of PGT-A is the embryo invasive biopsy procedure. The long-term bio-safety issue of embryo biopsy has not been investigated in humans (Desmyttere et al., 2012), whereas negative influences from maternal placenta to offspring neural and adrenal developments have been observed in mice (Zeng et al., 2013; Wu et al., 2014; Yao et al., 2016). Although several studies have shown that trophectoderm (TE) biopsy is not detrimental to reproductive competence in the blastocyst stage (Scott et al., 2012; Tiegs et al., 2021), some studies have reported that trophectoderm biopsy increases the rate of pregnancy loss and may negatively affect implantation rates (Guzman et al., 2019; Sonali et al., 2019). What is more, Zhang et al. (2019) have recently shown that TE biopsy is associated with a threefold increase in the risk of preeclampsia. Thus, is it possible that the biopsy could reduce the developmental potential and the implantation of the embryo while not affecting the potentiality of the embryo in terms of increasing obstetric or neonatal risks? There is no clear answer yet. The long-term potential risk to offspring safety should be considered. Therefore, a non-invasive and convenient approach would facilitate the widespread application of PGT, thereby improving success rates.

The existence of DNA in the blastocoel fluid was first reported by Palini et al. (2013), and a pilot study was conducted to perform chromosome screening using blastocentesis (Gianaroli et al., 2014). Genomic and mitochondrial DNA contents were discovered in the culture medium and might serve as potential DNA sources for PGT (Stigliani et al., 2013; Shamonki et al., 2016). Studies have reported the utilization of secreted genomic DNA in the culture medium for preimplantation genetic diagnosis (PGD) of X-linked disorders and α-thalassemia cases (Assou et al., 2014; Wu et al., 2015). However, Hammond et al. (2017) and Vera-Rodriguez et al. (2018) reported a mixed origin of DNA in spent medium with varied proportions of maternal contamination and Capalbo et al. (2018) found that spent medium only produced a diagnosis concordance of 20.8% with TE biopsy for monogenetic disorder, making the feasibility of using spent medium for non-invasive genetic assessment more controversial.

Recently, we reported a non-invasive chromosome screening (NICS) assay that incorporates a multiple annealing and looping-based amplification cycle (MALBAC)-NGS strategy conducted on the spent medium of IVF embryos, and we validated the assay versus the whole embryo in 42 embryos (Xu et al., 2016). Subsequently, some scholars have carried out repeated verification and clinical application (Zong et al., 2012; Fang et al., 2019; Huang et al., 2019; Jiao et al., 2019; Yeung et al., 2019). Moreover, there is a related large sample size multicenter prospective study in progress and the interim analysis was reported to include 1,301 blastocysts to assess consistency between embryo cell-free DNA from spent blastocyst medium (SBM) and trophectoderm (TE) biopsy (Rubio et al., 2020). However, comparisons with whole embryo euploidy are lacking. Here, in the present study, three different samples from the same embryo including conventional TE, the corresponding spent culture medium, and the remaining whole embryo from 265 donated human IVF embryos were collected separately and used for subsequent sequencing. We demonstrated the validation of the NICS approach in a larger set of embryos with the whole embryo as a gold standard. The performances of NICS and trophectoderm-biopsy-based PGT (TE-PGT) were compared and contrasted through mutual secondary validation to investigate the feasibility of NICS for prioritizing embryos in IVF treatments.

## Materials and Methods

### Study Design and Embryo Samples Preparation

**FIGURE 1:**
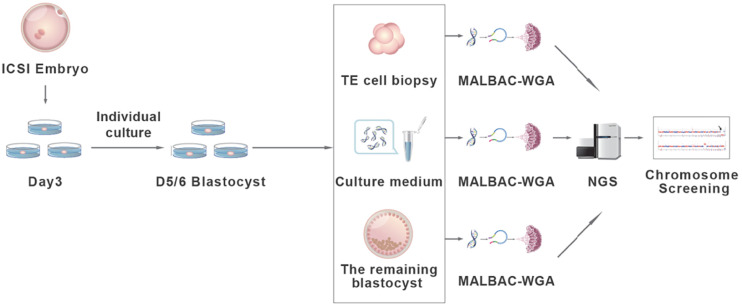
The validation procedure of the non-invasive chromosome screening (NICS) assay. Briefly, D3 embryos achieved *via* intracytoplasmic sperm injection (ICSI) were individually cultured in blastocyst culture medium and grow to blastocysts. D5/6 blastocysts were morphologically evaluated according to Gardner score. Blastocysts ineligible for transfer were used for subsequent analysis. Three to five trophectoderm cells (TE cells), the spent culture medium, and the remaining whole embryos were collected separately and used for whole genome amplification (WGA) by multiple annealing and looping-based amplification cycles (MALBAC). Whole-genome–amplified products were sequenced using an Illumina HiSeq 2500 platform and the chromosome ploidy information was obtained from all three assays for comparison.

The experimental design and procedure are illustrated in Figure 1. A total of 265 embryos were recruited from the Reproductive Medicine Centre of Affiliated Jinling Hospital, Medicine School of Nanjing University in 2017–2018. The ages of the female patients range from 24 to 39. IRB approvals (reference number 2016NJKY-028) and informed consents were obtained prior to analyzing the embryos. The registration number is ChiCTR-DDD-17010376 and the date is January 11, 2017.

**FIGURE 2:**
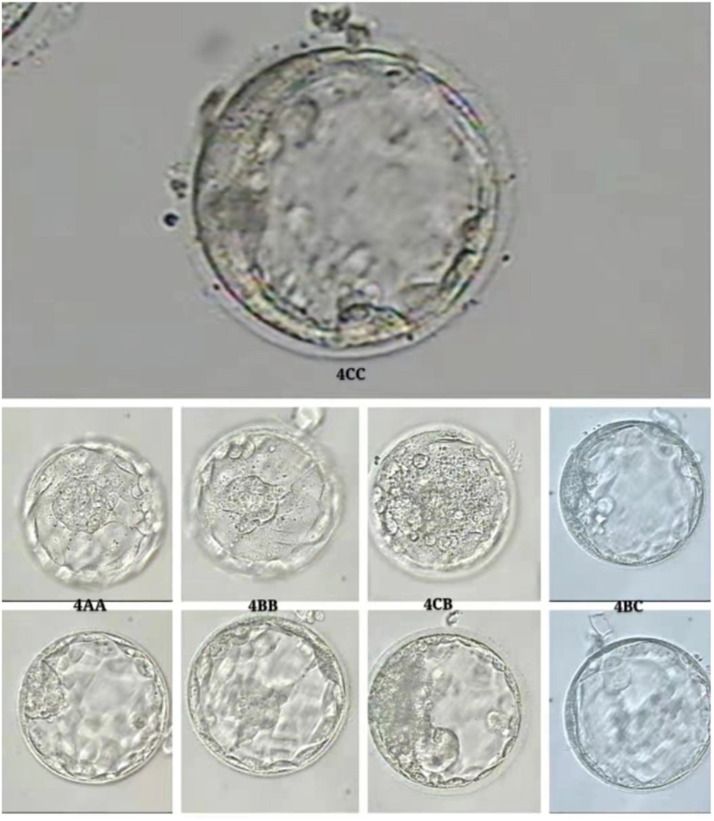
The assessment of embryo viability using blastocyst scoring system.

The embryos were all derived from intracytoplasmic sperm injection (ICSI) and cultured to cleavage stage (D3). Then, each embryo was rinsed and transferred into an individual drop of fresh blastocyst culture medium (SAGE, Cooper Surgical Fertility Co., Denmark). Blastocysts at D5/6 were then scored by morphological criteria according to a three-part scoring system developed by Gardner and Schoolcraft (1999) based on blastocyst expansion, the inner cell mass (ICM), and trophectoderm development before transfer. For blastocysts graded as 3–6, the development of the ICM was assessed as follows: A, tightly packed, many cells; B, loosely grouped, several cells; or C, very few cells. The TE was assessed as follows: A, many cells forming a cohesive epithelium; B, few cells forming a loose epithelium; or C, very few large cells. By using this scoring system, embryos scored 4CC were considered by the clinician to be not eligible for transfer. These embryos were donated and used for subsequent analysis (Figure 2).

Twenty to 25 μl of blastocyst media from each embryo was transferred into RNase–DNase-free PCR tubes each containing 5 μl of cell lysis buffer (Yikon Genomics, China) and stored at −80°C until use. The blank media, which had been exposed to procedures and conditions identical to those of the embryo media, were negative controls. To compare the NICS assay with the TE-PGT, three to five trophectoderm cells (TE cells) were biopsied from each of the blastocysts and transferred into 5 μl of cell lysis buffer. The remaining embryo cells from each blastocyst after the TE biopsy were also transferred into 5 μl of lysis buffer. The whole embryos were treated as the gold standard for this comparison study. The cell lysis was performed with 10 μl of culture media, TE biopsy, and the remaining whole embryo as described by the manufacturer (Yikon Genomics, China). Quantitative-PCR was conducted to quantify the cell-free DNA in blank and spent culture media.

### Whole Genome Amplification and Next-Generation Sequencing

The whole genome amplification (WGA) was performed on the media, TE cell biopsies, and whole embryos by MALBAC as described previously (Zong et al., 2012; Xu et al., 2016). Briefly, the amplification started with annealing the DNA to a pool of random primers. A quasi-linear pre-amplification step was performed before exponentially amplifying the DNA up to 2 μg. Agarose gel electrophoresis and quantification by Nano drop was conducted to evaluate the amplification efficiency. The amplified products were subjected to next-generation sequencing (NGS) library construction and sequenced on an Illumina HiSeq 2500 platform, yielding 2 million sequencing reads on each sample (Zong et al., 2012; Xu et al., 2016). The high-quality reads were extracted and mapped to the human hg19 genome. After removing duplication reads, the read numbers were counted along the whole genome with a bin size of 1 Mb and normalized by the GC content and a reference dataset to represent the relative copy number. The copy number of each bin was then segmented by circular binary segmentation (CBS) algorithms to merge bins with similar trends and calculate the final copy number segments. The coefficient of variation (CV), calculated as the ratio of the standard deviation of read density to its average, was used to assess the amplification success. A CV value of less than 0.2 was considered as a successful amplification. The R program was used to graph the copy number of each bin to visualize copy number variation (CNV) profiles for all 24 chromosomes. The NGS data for all embryo trios (biopsy, whole embryo, and spent culture media) have been deposited to the NCBI SRA database, and the BioProject accession number is PRJNA720213.

### Statistical Analysis

The performances of NICS and TE-PGT to select abnormal embryos by comparing with the CNV from the whole embryos were evaluated using sensitivity, specificity, negative predictive value (NPV), and positive predictive value (PPV) with 95% confidence intervals (CIs). The 95% CIs for the single proportions were calculated by the Wilson score method. The difference of the aforementioned diagnostic index between two assays was tested statistically by exact test for paired binary data. The concordance of different assays was analyzed by calculating Cohen’s Kappa coefficient and its 95% CI. A two-sided *p*-value of less than 0.05 was statistically significant. Statistical analyses were executed with SAS 9.4 (SAS Institute Inc., Cary, NC, United States).

## Results

### Performance of the NICS Assay to Discriminate Abnormal Embryos From Euploid Ones

**FIGURE 3:**
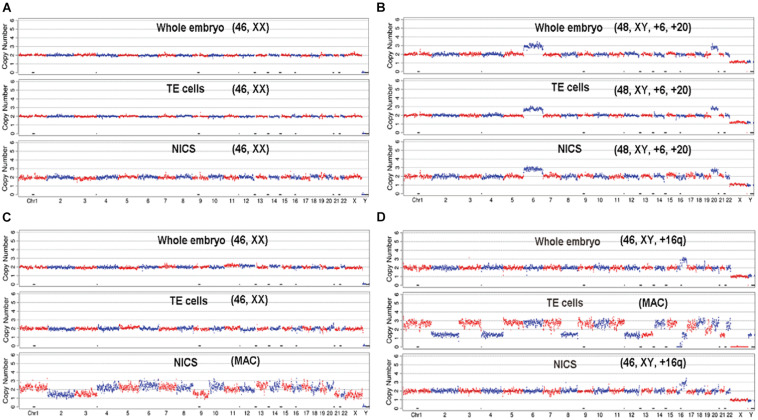
The demonstration of CNV profiles. The profiles generated from the NGS data of whole embryo, TE cells, and NICS were graphed and compared for each embryo. **(A)** The embryo shows consistent euploid karyotype of [46, XX] in all three assays. **(B)** The embryo shows consistent aneuploid karyotype of [48, XY, +6, +20] in all three assays. **(C)** The whole embryo and TE cells indicate a normal karyotype of [46, XX]; however, NICS shows multiple abnormal chromosomes (MAC). **(D)** The embryo shows consistent profile of [46, XY, +16q (q11.2 →qter, ∼47M)] in whole embryos and NICS assay; however, the TE cells show multiple abnormal chromosomes.

The average of DNA concentration quantified by Q-PCR was 0.423 ± 0.217 pg/μl in blank media and 6.090 ± 3.917 pg/μl in spent culture media. A total of 256 embryos out of 265 generated qualified NGS data for all three types of assays, which were used for the subsequent CNV analysis. The CNV patterns obtained from TE-PGT, NICS, and whole blastocyst embryos were graphed and compared (Figure 3 and Supplementary Table 1).

We treated the results from whole embryos as the gold standard to evaluate the performances of TE-PGT and NICS to discriminate abnormal embryos (aneuploid and mosaic) from euploid ones. We considered the results concordant if the two assays both generated chromosomal normal or chromosomal abnormal results. As shown in Table 1, compared with the whole embryo, the TE-biopsy-based PGT–CCS method yielded a performance of 89.6% (95% CI: 81.9–94.2%), 80.0% (95% CI: 73.1–85.5%), 92.8% (95% CI: 87.2–96.0%), and 72.9% (95% CI: 64.2–80.1%) for sensitivity, specificity, NPV, and PPV, respectively. Comparatively, the sensitivity and NPV of NICS assay were 86.5% (95% CI: 78.2–91.9%) and 90% (95% CI: 83.6–94.1%), respectively. However, the specificity [73.1% (95% CI: 65.8–79.4%)] and PPV [65.9% (95% CI: 57.2–73.6%)] showed slightly lower values compared with TE-PGT, but the difference was not statistically significant (Table 1, *p* > 0.05).

**TABLE 1:** The performance of TE-PGT versus NICS on detecting chromosomal abnormalities.

Embryo	*N*	Assay	Sensitivity % (95% CI)	Specificity % (95% CI)	NPV % (95% CI)	PPV % (95% CI)
All	256	TE-PGT	89.6 (81.9–94.2)	80.0 (73.1–85.5)	92.8 (87.2–96.0)	72.9 (64.2–80.1)
		NICS	86.5 (78.2–91.9)	73.1 (65.8–79.4)	90.0 (83.6–94.1)	65.9 (57.2–73.6)
		*p*	0.6291	0.1524	0.5144	0.2677

*The performance was assessed by comparing with the CNV from the whole embryo assay as the gold standard. The p-value represents the difference of diagnostic index between two assays. NPV, negative predictive value; PPV, positive predictive value.*

### The Chromosomal Composition in TE Biopsies, Spent Culture Media, and Whole Embryos

**FIGURE 4:**
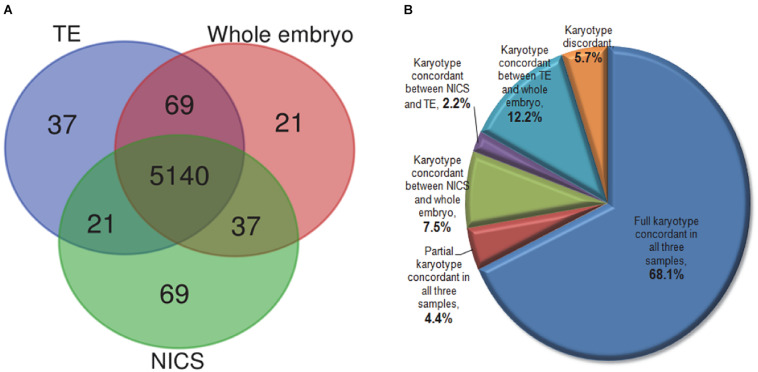
The comparison of karyotype in TE, NICS, and whole embryo samples. **(A)** The Venn diagram of the concordance of chromosome ploidy compared in three types of samples. The ploidy for all 22 autosomes and chromosome X in 229 non-MAC embryos (23 × 229 = 5,267) were compared among TE biopsy, NICS, and whole embryo. **(B)** The distribution of embryos with different karyotype concordances. Full concordance was reported if samples of two assays were euploid or if identical chromosomes were abnormal (including mosaic and/or reciprocal losses and gains); partial concordance was reported when at least one abnormal chromosome corresponded in both assays under comparison.

In order to evaluate the chromosomal composition in TE biopsies, spent culture media, and whole embryos, we also compared the ploidy per chromosome base in a total of 229 non-multiple abnormal chromosomes (MAC) embryos (Figure 4A). Out of 5,267 chromosome sets (each autosome or chromosome X of each embryo considered as one set), 5,140 (97.6%) showed consistent ploidy in all TE, NICS, and whole embryo specimens. NICS had 37 (0.7%) and 21 (0.4%) chromosomes consistent solely with TE and the whole embryo, respectively, and 69 (1.3%) were different from both.

As displayed in Figure 4B, 72.5% of embryos share concordant karyotypes in all three types of samples. We observed concordant karyotypes only in NICS versus the whole embryo but different in TE in 7.5% of embryos. A small proportion (2.2%) had consistent karyotypes in NICS and TE but not with the whole embryo. The TE and the whole embryo had concordant karyotypes in 12.2% of embryos, which, however, had distinctive karyotypes in NICS.

### Embryos With Multiple Abnormal Chromosomes

In our ploidy analyses, embryos with five or more abnormal chromosomes were defined as carrying MAC. Notably, if embryos identified with MAC by TE-PGT (accounting for 3.9% of the total embryos) were excluded from the dataset, the specificity and the PPV of TE-PGT assay increased slightly to 82.6% (95% CI: 75.8–87.7%) and 75.0% (95% CI: 66.1–82.2%), respectively (Table 2). On the other hand, the specificity and the PPV of NICS assay also improved prominently to 79.6% (95% CI: 72.4–85.3%) and 72.2% (95% CI: 63.1–79.8%) after the exclusion of 18 embryos identified with MAC by NICS (7.0% of all embryos). Furthermore, both assays showed improved performance after the exclusion of all MAC embryos identified by either assay (Table 2).

**TABLE 2:** The performance of NICS versus TE-PGT on detecting chromosomal abnormalities after the exclusion of MAC embryos.

Embryo	*N*	Assay	Sensitivity % (95% CI)	Specificity % (95% CI)	NPV % (95% CI)	PPV % (95% CI)
MAC embryos identified by TE-PGT excluded	246	TE-PGT	89.0 (80.9–93.9)	82.6 (75.8–87.7)	92.8 (87.2–96.0)	75.0 (66.1–82.2)
MAC embryos identified by NICS excluded	238	NICS	85.7 (77.1–91.5)	79.6 (72.4–85.3)	90.0 (83.6–94.1)	72.2 (63.1–79.8)
MAC embryos identified by PGT/NICS excluded	229	TE-PGT	89.7 (81.5–94.5)	82.4 (75.3–87.8)	92.9 (87.0–96.2)	75.7 (66.6–83.0)
		NICS	87.4 (78.8–92.8)	80.3 (73.0–86.0)	91.2 (84.9–95.0)	73.1 (63.8–80.7)
		*p*	0.7905	0.7359	0.6495	0.7505

*The performance was assessed by comparing with the CNV from the whole embryo assay as the gold standard. NPV, negative predictive value; PPV, positive predictive value; MAC, multiple abnormal chromosomes.*

The concordance of chromosomal ploidy between NICS/TE-PGT versus the whole embryo was investigated by Kappa analysis and demonstrated in Table 3. In all 256 embryos, the TE-PGT showed a concordance of 83.6% (95% CI: 78.6–87.6%) (Kappa = 0.665, 95% CI: 0.574–0.756) with the gold standard (whole embryo), while NICS had a concordance of 78.1% (95% CI: 72.7–82.8%) (Kappa = 0.561, 95% CI: 0.462–0.660). The exclusion of MAC embryos also resulted in improvements in the concordance between TE-PGT/NICS versus the whole embryo, generating a concordance of 85.2% (95% CI: 78.0–89.2%) (Kappa = 0.696, 95% CI: 0.602–0.789) for TE-PGT and 83.0% (95% CI: 77.6–87.3%) (Kappa = 0.652, 95% CI: 0.553–0.750) for NICS by comparing with the standard whole embryo (Table 3).

**TABLE 3:** The Kappa concordance analysis between different assays.

Embryo			Whole embryo			Concordance % (95% CI)	Kappa (95% CI)
			+	−	Total		
All	TE-PGT	+	86	32	118	83.6 (78.6–87.6)	0.665 (0.574–0.756)
		−	10	128	138		
		Total	96	160	256		
	NICS	+	83	43	126	78.1 (72.7–82.8)	0.561 (0.462–0.660)
		−	13	117	130		
		Total	96	160	256		

			**TE-PGT**			**Concordance % (95% CI)**	**Kappa (95% CI)**
			**+**	**−**	**Total**		

	NICS	+	89	37	126	74.2 (68.5–79.2)	0.484 (0.377–0.591)
		−	29	101	130		
		Total	118	138	256		

**MAC embryos identified by PGT/NICS excluded**			**Whole embryo**			**Concordance % (95% CI)**	**Kappa (95% CI)**
			**+**	**−**	**Total**		

	TE-PGT	+	78	25	103	85.2 (78.0–89.2)	0.696 (0.602–0.789)
		−	9	117	126		
		Total	87	142	229		
	NICS	+	76	28	104	83.0 (77.6–87.3)	0.652 (0.553–0.750)
		−	11	114	125		
		Total	87	142	229		

			**TE-PGT**			**Concordance % (95% CI)**	**Kappa (95% CI)**
			**+**	**−**	**Total**		

	NICS	+	79	25	104	78.6 (72.8–83.4)	0.568 (0.461–0.675)
		−	24	101	125		
		Total	103	136	229		

*MAC, multiple abnormal chromosomes.*

### Categorizing and Scoring Embryos According to NICS and TE-PGT Results

The embryos can be categorized or scored by the obtained NICS results into three groups: (A) chromosomal normal, (B) chromosomal abnormal, and (C) MAC or uncertain (Table 4). Out of the 256 embryos with conclusive outcomes, 130 embryos (50.8%) were classified into the chromosomal normal group, in which 117/130 (90.0%) were consistently normal according to the gold standard whole embryo; 108 embryos (42.2%) were classified into the chromosomal abnormal group, of which 30/108 (27.8%) were normal based on the whole embryo assay. Among the 18 embryos (7.0%) classified into the MAC group, 13 (72.2%) were chromosomal normal according to the whole embryo. On the other hand, TE-PGT also classified embryos into three categories in a similar manner (Table 4): 138 (53.9%) were chromosomal normal (group A) with 128 (92.8%) normal according to the whole embryo standard; 108 embryos (42.2%) were classified into chromosomal abnormal (group B), of which 27/108 (25.0%) were normal based on the standard; and 10 (3.9%) embryos were categorized into group C, 5 of which (50.0%) were defined as normal by the standard.

**TABLE 4:** Categorizing embryos according to NICS and TE-PGT results.

	Total	Group A		Group B		Group C	
NICS	256	130 (50.8%)		108 (42.2%)		18 (7.0%)	
		TN	FN	TP	FP	TP	FP
		117 (90.0%)	13 (10.0%)	78 (72.2%)	30 (27.8%)	5 (27.8%)	13 (72.2%)
TE-PGT	256	138 (53.9%)		108 (42.2%)		10 (3.9%)	
		TN	FN	TP	FP	TP	FP
		128 (92.8%)	10 (7.2%)	81 (75.0%)	27 (25.0%)	5 (50.0%)	5 (50.0%)

*The result of the whole embryo assay was treated as the reference. **(A)** Chromosomal normal; **(B)** chromosomal abnormal; **(C)** MAC or uncertain. TN, true negative; FN, false negative; TP, true positive; FP, false positive compared to the whole embryo assay.*

Interestingly, in the MAC group of NICS, if the outcomes from an addition of TE-PGT assay were also considered for further confirmation, the accuracy would have been increased to 83.3% (15/18) instead of 27.8% (5/18) by NICS alone. In particular, 12/18 (66.7%) of the MAC embryos could have been re-categorized into the chromosomal normal group by additional TE-PGT (Table 5). Similarly, in the abnormal group of NICS, a secondary confirmation by TE-PGT could increase the accuracy from 72.2% (78/108) to 84.3% (91/108) with 25/108 (23.1%) embryos re-categorized into the normal group (Table 6).

**TABLE 5:** Characteristics of embryos with results of MAC (group C) obtained from NICS or TE-PGT assay.

Assay	No. of embryos with MAC results by NICS/TE-PGT						
	Total	Abnormal by whole embryo			Normal by whole embryo		
NICS	18	Total (%)	Abnormal by TE-PGT (%)	Normal by TE-PGT (%)	Total (%)	Normal by TE-PGT (%)	Abnormal by TE-PGT (%)
		5 (27.8%)	4 (22.2%)	1 (5.6%)	13 (72.2%)	11 (61.1%)	2 (11.1%)
TE-PGT	10	Total (%)	Abnormal by NICS (%)	Normal by NICS (%)	Total (%)	Normal by NICS (%)	Abnormal by NICS (%)
		5 (50.0%)	3 (30.0%)	2 (20.0%)	5 (50.0%)	3 (30.0%)	2 (20.0%)

*The result of the whole embryo assay was treated as the reference. MAC, multiple abnormal chromosomes.*

**TABLE 6:** Characteristics of embryos with results of chromosomal abnormalities (group B) obtained from NICS or TE-PGT assay.

Assay	Number of embryos with chromosomal abnormal results by NICS/TE-PGT						
	Total	Abnormal by whole embryo			Normal by whole embryo		
NICS	108	Total (%)	Abnormal by TE-PGT (%)	Normal by TE-PGT (%)	Total (%)	Normal by TE-PGT (%)	Abnormal by TE-PGT (%)
		78 (72.2%)	72 (66.6%)	6 (5.6%)	30 (27.8%)	19 (17.6%)	11 (10.2%)
TE-PGT	108	Total (%)	Abnormal by NICS (%)	Normal by NICS (%)	Total (%)	Normal by NICS (%)	Abnormal by NICS (%)
		81 (75.0%)	73 (67.6%)	8 (7.4%)	27 (25.0%)	16 (14.8%)	11 (10.2%)

*The result of the whole embryo assay was treated as the reference.*

## Discussion

### Design and Limitation of the Current Study

Because PGT involves invasive embryo biopsies and TE biopsy may not truly represent the chromosomal status of ICM, which develops into the fetus, the universal use of the conventional TE-PGT in ART has been controversial. Animal studies have shown that the source of cell-free DNA from apoptotic cells in culture fluid mainly originated from the ICM, which is more representative of the fetus than TE biopsied cells (Bolton et al., 2016). Therefore, we previously developed a spent-culture-medium-based PGT method named NICS and applied the technique on patients with abnormal karyotype, repeated implantation failure, and recurrent pregnancy loss (RPL) (Xu et al., 2016; Fang et al., 2019). By applying an appropriate threshold for mosaicism, NICS was less prone to errors associated with embryo mosaicism and was more reliable than TE biopsy PGT-A (Huang et al., 2019). Are the results of chromosome test analysis using SCM more representative of the genetic traits of whole embryos? In the present study, by using the whole embryo as a gold standard, we performed the NICS validation test in a much larger set of embryos [256 embryos versus 42 embryos previously (Xu et al., 2016)]. For each embryo, we have tested both the spent culture medium and trophectoderm biopsy sample (while in the earlier study, we have only tested the spent culture media but not the trophectoderm biopsies) and compared the test results to the whole embryo, hoping to provide more solid data for the technology to be used clinically and more insights into the origin of the DNA molecules in culture medium.

In general, NICS exhibits a comparable accuracy in detecting chromosomal abnormality compared to TE-PGT (Table 1) with a promising NPV of >90%, which is consistent with the previous publication (NPV 91.3%) (Xu et al., 2016). The specificity and PPV were 80.0 and 72.9% for TE-PGT and 73.1 and 65.9% for NICS, comparable to the specificity of 84.0% and PPV of 78.9% of NICS in the previous report. We attribute the lower PPV in the current study mainly to the fact that we are using embryos with lower morphology scores that were not eligible for transfer. These embryos may have higher incidence of debris and DNA fragmentation compared with the embryos eligible for transfer, which may contribute to the increased false positives observed in this study.

### The Potential Contamination in the Spent Culture

It has been raised that the potential sources of contamination in spent culture media include the background genetic material in the medium, contamination during the culture period, and the presence of maternal contamination derived from cumulus cells (Hammond et al., 2016, 2017). In the present study, we included blank media, which were cultured under the same condition with the embryos, as negative controls. A significantly lower baseline DNA quantity compared with the spent culture medium (0.423 versus 6.09 pg/μl) suggested a limited impact of background contamination on NICS outcomes. False negative is thought to be partially attributed to the maternal originated contamination such as cumulus cells, which have a balanced chromosomal content (Xu et al., 2016; Feichtinger et al., 2017; Vera-Rodriguez et al., 2018). The high discordant rate (67%) of the spent culture medium result with TE biopsies is mainly due to the high percentage of maternal DNA in the culture media (Vera-Rodriguez et al., 2018). However, in our datasets, we only observed 13 false negatives, which all harbor concordant sex chromosomes in NICS compared with whole embryos and TE. A recent investigation from another group (Ho et al., 2018) reported a concordance of 81.3% for ploidy between D3 spent culture medium and whole embryo and a concordance of 70% between D5 spent culture medium and TE biopsy, which is quite consistent with our data (Table 3 and Figure 4B). The low proportion of maternal contamination and higher concordant rate observed in the present study compared with Vera-Rodriguez et al. (2018) might be attributed to three aspects: (a) thorough precautions were taken to remove as many cumulus–corona radiata cells as possible including rinsing the embryos repeatedly when changing the medium on day 3. (b) We utilized the MALBAC WGA technique, which has been reported to have better uniformity on amplification and to be suitable for CNV analysis (Huang et al., 2015; He et al., 2018). However, Vera-Rodriguez et al. (2018) employed a double WGA strategy, which has a higher chance of introducing uneven amplification. (c) We used the whole embryo instead of TE biopsy as the gold standard for comparison. The karyotype of TE cells does not necessarily represent that of whole embryos. As shown in Figure 4B, 7.5% of embryos had different karyotypes in TE versus whole embryos and NICS, most probably due to mosaics. Therefore, the concordance of NICS compared with TE biopsy is conceivably lower than that compared with whole embryos (Table 3).

### NICS Provides a Scoring System Based on Probability of Ploidy Abnormalities for Prioritizing Embryos for Transfer

We note that based on the NICS result of chromosomal normal, abnormal, or MAC, embryos can be categorized into three groups, each predicting a different ploidy normal probability in the whole embryo. Group A predicts a normal embryo with 90.0% probability, while groups B and C predict 27.8 and 72.2% normal probability, respectively (Table 4). The distinct ploidy normal probabilities predicted by the three groups show that the NICS assay is effective in providing a euploidy-based scoring system for prioritizing embryo for transfer, thereby potentially improving implantation per transfer and reducing spontaneous abortion due to aneuploidy. The high NPV of NICS shows that the assay is a good rule-in assay for identifying euploid embryo for transfer. However, even in group B, the embryos still have a 27.8% chance of being normal, showing that NICS is less effective in ruling out aneuploid embryos for transfer. However, when an addition of TE-PGT assay is provided as a secondary validation in groups B and C, the accuracy of detection greatly increases to >80% in both groups (Tables 5, 6).

We suggest that embryos in group A should be prioritized in embryo transfer. If only group B or C embryos are obtained, invasive biopsy-based PGT might be performed for further characterization. This would generally require a subsequent biopsy on frozen thawed embryos as well as a second vitrification-warming to allow sufficient time for genetic testing, which has been reported to reduce the clinical pregnancy rate slightly (Bradley et al., 2017). However, we have developed a pipeline for quick NICS and TE-PGT, and the whole procedure can be accomplished within 9 h. Therefore, NICS can be performed in time for fresh blastocyst biopsy on later D5 or earlier D6, or biopsy-based PGT on frozen thawed embryos might be done in time for subsequent transfers without additional vitrification-warming. Alternatively, embryos (in group B or C) may be selected to transfer based on their morphology score to avoid falsely ruling out euploid embryos.

In fact, based on the result of invasive TE-PGT, embryos can also be categorized into three groups, with a chromosomal normal prediction probability of 92.8, 25.0, and 50.0% for groups A, B, and C, respectively, which is actually not significantly better than the 90.0, 27.8, and 72.2%, by NICS (Table 4). Therefore, invasive TE-PGT is also an effective rule-in assay for selecting euploid embryos but is less effective in ruling out embryos, which is consistent with the finding that transferring mosaic or abnormal embryos predicted by PGT-A still result in successful pregnancies and live births (Greco et al., 2015).

### NICS May Provide an Insight Into Whether Mosaic Embryos Detected by TE-PGT Should Be Transferred

Mosaic has been a hotly debated issue of PGT-A, which makes universal use of PGT-A highly controversial in the past few years (Cohen et al., 2013; Elpida et al., 2013; Gleicher et al., 2017). The presence of mosaicism has further affected the reliability and accuracy of using a single biopsy of only 5–10 TE cells. The whole embryo analysis should be acknowledged as having less opportunity to have mosaicism identified since complementary abnormalities from mitotic non-disjunction would be balanced out and appear normal when evaluating all the cells in one sample. Theoretically, because the genetic material is released into culture media from both ICM and TE cell lineages, NICS can more comprehensively reflect the genetic traits of embryos, so the embryos that were diagnosed mosaic after NICS will increase. In fact, the mechanism(s) underlying mosaicism remains elusive, and the genetic material obtained from the spent culture medium of mosaic embryos is undefined. However, NICS can be used either as a preliminary screening strategy, and then only embryos with positive results were biopsied, or as a way to provide an insight into whether mosaic embryos detected by TE-PGT should be transferred.

**FIGURE 5:**
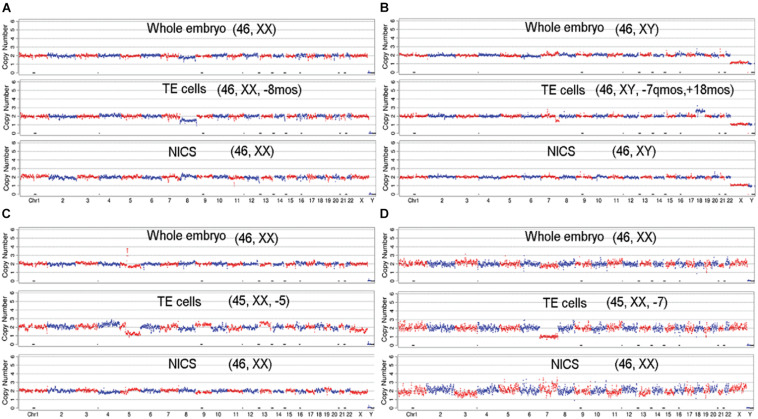
The CNV profiles of mosaic embryos with aneuploid/mosaic aneuploid TE biopsies, but with euploidy in whole embryo and NICS. The embryos show normal karyotype in whole embryo and NICS, but TE cells indicate mosaic profiles: **(A)** [46, XX, –8mos]; **(B)** [46, XY, –7q(q31.33 → qter, ∼30M) mos, +18mos]; or aneuploid profiles: **(C)** [45, XX, –5]; **(D)** [45, XX, –7].

Dynamic genetic self-correction during the embryo growth has been reported (Kyle et al., 2015; Lagalla et al., 2017; Lee and Kiessling, 2017) and mosaic aneuploid embryos transferred also showed the potential to develop to healthy euploid newborns (Greco et al., 2015; Inoue et al., 2017). Therefore, for women with mosaic embryos but no euploid embryos, the selection of mosaic embryos for ET might be an option. In such cases, the PGT-A results might be misleading because the TE biopsy does not often represent the constitution of whole embryos especially the inner cells (Gleicher et al., 2017). Alternatively, NICS targeting the cell-free DNA secreted in the medium might be a supplementary strategy. As shown in Figure 4, 37 (0.7%) chromosome sets show concordant ploidy only between NICS and whole embryo specimens and 7.5% of blastocysts with whole embryo chromosomal composition are representative of NICS instead of TE, which is probably due to mosaicism. Interestingly, in the present study, we discovered 19 embryos that were identified as aneuploid or aneuploidy mosaic by TE-PGT (16 in group B and 3 in group C), but indicated as normal karyotype by both NICS and the whole embryo. The CNV profiles of some examples are illustrated in Figure 5. Conceivably, instead of preventing transfer of aneuploid embryos, utilization of NICS as an adjunct approach to conventional TE-PGT might assist in more efficiently screening mosaic embryos potentially eligible for transfer. For instance, the mosaic embryo with a mosaic monosomy of chromosome 8 in TE biopsies (Figure 5A) might be considered for transfer according to the recent recommendation by the Preimplantation Genetic Diagnosis International Society (PGDIS).

In conclusion, this is the first large-scale (265 embryos) validation study to investigate NICS performance versus invasive TE-PGT and the whole embryo ploidy. Our results suggest that NICS is a good rule-in assay for identifying chromosomal normal embryos for transfer and might serve as a non-invasive approach prior to invasive TE-PGT for prioritizing embryos for transfer. We envision that with further clinical studies and validations, NICS might provide a safer alternative in embryo screening for improving clinical outcomes of assisted reproductive technology.

## Data Availability Statement

The data presented in the study are deposited in the NCBI SRA repository, accession number PRJNA720213.

## Ethics Statement

The studies involving human participants were reviewed and approved by Ethics Committee of Nanjing General Hospital, Nanjing Military Region. The patients/participants provided their written informed consent to participate in this study. Written informed consent was obtained from the individual(s) for the publication of any potentially identifiable images or data included in this article.

## Author Contributions

BY: study design and final version of manuscript approval. JX, HF, and YL: data acquisition. YY and SL: data analysis and interpretation. LC and QS: drafting manuscript and responsible for the integrity of the data analysis. All authors contributed to the article and approved the submitted version.

## Conflict of Interest

YY and SL were employed by company Yikon Genomics Company, Ltd. The remaining authors declare that the research was conducted in the absence of any commercial or financial relationships that could be construed as a potential conflict of interest.

## Publisher’s Note

All claims expressed in this article are solely those of the authors and do not necessarily represent those of their affiliated organizations, or those of the publisher, the editors and the reviewers. Any product that may be evaluated in this article, or claim that may be made by its manufacturer, is not guaranteed or endorsed by the publisher.
